# Tri-Variate Relationships among Vegetation, Soil, and Topography along Gradients of Fluvial Biogeomorphic Succession

**DOI:** 10.1371/journal.pone.0163223

**Published:** 2016-09-20

**Authors:** Daehyun Kim, John A. Kupfer

**Affiliations:** 1 Biogeomorphology Research and Analysis Group, Department of Geography, University of Kentucky, Lexington, KY, 40506, United States of America; 2 Department of Geography, University of South Carolina, Columbia, SC, 29208, United States of America; University of California Davis, UNITED STATES

## Abstract

This research investigated how the strength of vegetation–soil–topography couplings varied along a gradient of biogeomorphic succession in two distinct fluvial systems: a forested river floodplain and a coastal salt marsh creek. The strength of couplings was quantified as *tri-variance*, which was calculated by correlating three singular axes, one each extracted using three-block partial least squares from vegetation, soil, and topography data blocks. Within each system, tri-variance was examined at low-, mid-, and high-elevation sites, which represented early-, intermediate-, and late-successional phases, respectively, and corresponded to differences in ongoing disturbance frequency and intensity. Both systems exhibited clearly increasing tri-variance from the early- to late-successional stages. The lowest-lying sites underwent frequent and intense hydrogeomorphic forcings that dynamically reworked soil substrates, restructured surface landforms, and controlled the colonization of plant species. Such conditions led vegetation, soil, and topography to show discrete, stochastic, and individualistic behaviors over space and time, resulting in a loose coupling among the three ecosystem components. In the highest-elevation sites, in contrast, disturbances that might disrupt the existing biotic–abiotic relationships were less common. Hence, ecological succession, soil-forming processes, and landform evolution occurred in tight conjunction with one another over a prolonged period, thereby strengthening couplings among them; namely, the three behaved in unity over space and time. We propose that the recurrence interval of physical disturbance is important to—and potentially serves as an indicator of—the intensity and mechanisms of vegetation–soil–topography feedbacks in fluvial biogeomorphic systems.

## Introduction

There is a growing recognition of the reciprocal nature of vegetation–environment relationships [1–5]. For example, riparian vegetation controls the cohesiveness, erosion, and transport of sediments, thereby affecting the size, shape, and stability of fluvial landforms, as well as the geochemistry of river bank soil substrates [6–8]. These abiotic components then feed back to determine habitat conditions and constrain the subsequent plant species interactions and vegetation dynamics through time. Therefore, vegetation, soil, and landforms are increasingly considered to behave as an intertwined complex that is materialized by their mutual interactions under the combined operation of allogenic and autogenic processes [9–11]. These ideas have been conceptualized as, to mention just a few, ‘ecosystem engineers’ that actively modify their surrounding physical environment [12–14], ‘niche construction’ by which such modifications increase the engineers’ chance of survival [15,16], and ‘biogeomorphic ecosystems’ in which habitat settings, species assemblages, and matter/energy fluxes are all emergent properties of organism–environment feedbacks [17]. This new biogeomorphic paradigm has been manifested especially at the land–water interface where dynamic hydrological processes drive the active motion of water, air, and sediment, and accelerate the interactions between these physical factors and vegetation cover [18–22].

**Fig 1:**
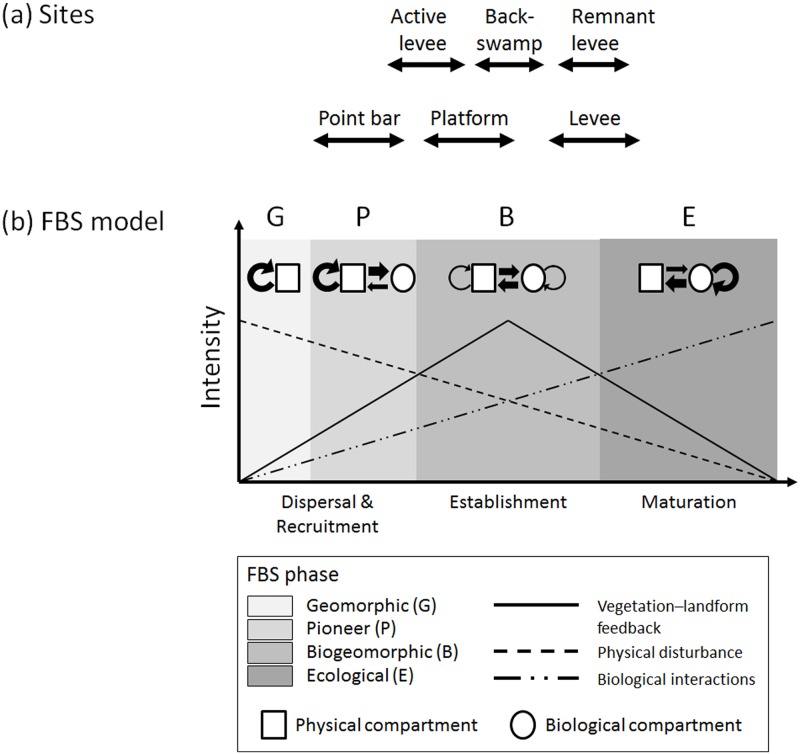
Fluvial biogeomorphic succession model. (a) Approximate placement of the six sites of two study areas (floodplain forest and salt marsh) along the gradient of fluvial biogeomorphic succession (FBS), (b) Simplified version of the FBS model developed by Corenblit *et al*. [10,23].

The purpose of this paper is to investigate how the strength of vegetation–soil–topography couplings varies along the gradient of the fluvial biogeomorphic succession (FBS) model, originally developed by Corenblit *et al*. [10,23]. The model consists of four stages of biogeomorphic succession (i.e., geomorphic, pioneer, biogeomorphic, and ecological), each being characterized by a specific suite of interrelations between plant species and landform substrate (Fig 1b). The first stage (the *geomorphic stage*) involves post-disturbance rejuvenation (e.g., following high-magnitude flooding: [24]) where the formation and stability of landforms are determined primarily by sediment cohesiveness and hydrodynamic forces that control the dispersal of plant diaspores [25,26]. In the *pioneer stage*, these diaspores establish and germinate on newly created bare surfaces. Geomorphology still exerts dominant allogenic controls on the survival and growth of seedlings [27–29]. During the subsequent *biogeomorphic phase*, vegetation–environment feedbacks occur through the tight interplay between the biomechanical traits of plants and the cohesiveness and shape of the substrate, which is mediated by the hydrogeomorphic flows of matter and energy [18,30]. The final stage (the *ecological stage*) is characterized by a disconnection from hydrogeomorphic disturbances, fluvial landform stabilization, and autogenic succession (i.e., biotic interactions dominate). This successional sequence can be completely or partially reinitiated at any time depending on the magnitude and/or frequency of major disturbance [31,32]. The potential efficacy of the FBS model has been discussed in a wide range of systems, encompassing upland rivers [33–35], salt marshes [11,17], and lateral moraines [36]. The model, however, has not been dealt with in the context of the strength of vegetation–substrate relationships based on field data.

Given the distinct regimes of hydrogeomorphic disturbance among the four successional phases, we predict that vegetation–soil–topography relationships are likely weak in the early FBS stages and become stronger as succession proceeds toward later stages. In the early geomorphic and pioneer phases, the system’s substrate is generally dynamic and unstable due to the short recurrence interval of physical disturbance (Fig 1b). Under such conditions, seedling recruitment and soil-forming processes are highly stochastic [37], and time between disturbances is insufficient for the establishment of significant couplings among vegetation, soil, and topography. In the later biogeomorphic and ecological stages, a tighter coupling is a more probable outcome due to greater substrate stability resulting from the longer disturbance recurrence interval. At these later stages, the vegetation–substrate complex is shaped increasingly by gradual, autogenic interactions among vegetation, soil, and topography, rather than by allogenic forces and randomness, which were predominant in earlier previous stages [38,39]. These predictions about the changing correlation structure between species and environment, although appearing intuitive, have rarely been empirically tested along fluvial disturbance or successional gradients. In this paper, we address this knowledge gap along the FBS gradient for two distinct fluvial systems: a bottomland hardwood floodplain forest and a coastal salt marsh creek. We posit that this research could serve as a springboard for answering a broader, long-standing question in ecology and Earth sciences: Does increasing biogeomorphic stability (i.e., coevolution of vegetation and substrate toward stabilization) lead to a *unified* or *synchronized* behavior of biological and physical components over space? (see [40]).

Vegetation, soil, and topography are multivariate in nature. In many cases, ecologists examine the abundance of multiple (*l*) plant species across a number of plots. In these same plots, they also acquire a set of soil data, composed of *m* physical and chemical properties, as well as topographic data of *n* landform parameters. To correlate these three distinct data domains (or blocks), we employ three-block partial least squares (3-B PLS), which is a recent multi-block technique that treats data blocks at the same level (i.e., symmetrical approach), rather than dividing them into the groups of independent and dependent variables as in the conventional regression framework [41]. The method, when extracting major components from a certain block, seeks simultaneously to maximize the explained variance of the other blocks. Therefore, the components originating from different blocks possess the greatest mutual linear predictive power [42–44]. Although PLS has been underrepresented in ecological literature, there is a growing body of researchers who demonstrate the usefulness of the approach, given its inherent design to overcome the typical problems in ecological data: (1) the number of independent variables is greater than the number of observations and/or (2) environmental variables are significantly correlated with one another (e.g., [45,46]).

## Materials and Methods

### Data collection

A brief explanation of the two study areas and data acquisition is presented below. More detailed information can be found in the supporting information of this paper (S1 Text, S1 and S2 Figs).

The floodplain data set comes from a study of post-logging succession at the Bates Fork tract within Congaree National Park, South Carolina, USA [47]. A total of 63 sample locations were established at sites: (1) adjacent to the active natural levee of the Congaree River (*n* = 31), (2) in backswamp locations of the floodplain interior between the current and abandoned levees (*n* = 14), and (3) on and adjacent to a remnant natural levee along Bates Old River, an abandoned channel of the Congaree River (*n* = 18) (S1 Fig). At each sample location, plant species composition was recorded in three circular plots (2.82 m radius) spaced evenly along a 30 m transect by measuring the diameter at breast height (DBH, 1.37 m) of all individual stems with DBH > 1 cm (S1 Fig). Within each plot, four soil cores were taken from the top 15 cm of forest floor, composited, dried, and analyzed for pH, extractable phosphorus, potassium, calcium, magnesium, zinc, copper, sodium, organic matter, cation exchange capacity (CEC), exchangeable acidity (the amount of total CEC occupied by H^+^ and Al^3+^), and total percent base saturation (the percent of exchange sites occupied by base cations). For each plot, two variables related to topography, and by extension, flood regime, were determined and averaged by location: (1) elevation, which was extracted from a high-resolution, LiDAR-derived digital terrain model for the study area, and (2) the depth of inundation during a 98,000 cfs flood, which was estimated using a flood inundation model (HEC-RAS) [47]. Our application to conduct research at Congaree National Park was submitted to Bill Hulslander (Chief of Resources and Science at the park) and approved by the National Park Service. Data collection did not involve endangered or protected species.

The study marsh is located at the Skallingen peninsula in southwestern Denmark (S2 Fig) and shows a complex network of tidal channels, most actively migrating through bar deposition and cutbank erosion across the marsh’s platform [48]. In the summer of 2006, we selected 11 point bars that were considered more or less representative of the cross-channel topographies at the marsh in terms of creek width, sinuosity, and depth (S2 Fig). From each bar, a line transect (ca. 25 m in length) was established perpendicular to the streamline. The transect fully encompassed three distinct topographic zones: newly deposited point bar, bank levee, and inner marsh field (or marsh interior). Along each transect, a total of 9–10 locations were chosen for soil and topographic surveys. Soil properties examined were soil pH, bulk density, electrical conductivity, nitrate, phosphorus, potassium, calcium, magnesium, sulfur, and sodium. Also, two topographic variables were identified: surface elevation and distance to the channel. In this case, distance was regarded as a proxy for sedimentation rate [49,50]. At each location, two replicate square quadrats (1 m × 1m, each subdivided into 25 grids) were established for recording the frequency of each vascular plant species present. The frequencies, varying from 0 to 25, from these quadrats were then averaged. Overall, species frequency data have been collected from 102 plots across the three sites: 26 in point bar, 41 in marsh platform, and 35 in bank levee. No specific permissions were required for these research activities at Skallingen, because the marsh was a public area. Data collection at the field did not involve endangered or protected species.

### Placing study sites along the FBS gradient

The three Congaree River floodplain sites captured a gradient of conditions related to differences in surface elevation, hydroperiod, geomorphic setting, and distance to the main-stem channel (see S1 Text, S1 and S2 Tables for more details). All three were clear cut at roughly the same time and were undergoing post-logging succession, albeit at different rates and along different successional trajectories [47]. Assuming that these among-site environmental heterogeneities have influenced the rate of vegetation dynamics, we differentiated the three sites along the FBS gradient as follows. Locations at the active levee site were situated on a meander scroll complex just beyond the margin of the currently active natural levee (S1 Fig). These sample locations were at the lowest elevations, closest to the main river, and experienced the greatest level of flood-associated disturbance. Vegetation was dominated by early-successional species, with the greatest component of shrubs and vines of any of the three sites. Given the hydrogeomorphic setting, substrate instability, and vegetational characteristics, we determined that the active levee site could be placed somewhere between the late-pioneer and the early-biogeomorphic phases (Fig 1a). The remnant levee site was on the natural levee of a meander bend which had been abandoned in the 1850s. This site was at the highest elevation and, thus, underwent the least frequent inundation that might occasionally induce overbank sedimentation. Regrowth was dominated to a much greater degree by regeneration of mid- to late-successional species (e.g., *Quercus* and *Carya*) more commonly associated with upland and transitional systems than at the active levee site. Considering the long time since abandonment, substrate stability, and anticipated maturity, this system could be best placed in the late-biogeomorphic or the early-ecological stage. The hydrogeomorphic conditions (i.e., elevation, hydroperiod, and disturbance regime) and ecological communities of the backswamp site in the floodplain interior were intermediate between those of the current and abandoned levees. Therefore, we posited that this site represented the intermediate-biogeomorphic stage.

Among the three site types of the Skallingen salt marsh (i.e., point bar, platform, and levee), the point bar was at the lowest elevation and experienced the most frequent and deepest submergence by sea water during high tidal flows. Furthermore, this was where dynamic reworking and deposition of sediments happened to modify the existing landforms and substrate availability. Only those early-successional species (e.g., *Spartina anglica*, *Puccinellia maritima*, *Salicornia herbacea*, and *Suaeda maritima*) that were tolerant to such unstable conditions could colonize and reproduce at point bars [11,51]. Hence, we designated the point bars of Skallingen as belonging to the pioneer stage of the FBS (Fig 1a). By contrast, natural levees were situated at the highest elevation and most free from the direct impacts of fluvial–geomorphic creek processes. Their stable substrate allowed the establishment of high-marsh plants such as *Festuca rubra*, *Artemisia maritima*, and *Juncus gerardii*. We considered that the levees could be placed somewhere from the late-biogeomorphic to the early-ecological phases of the FBS. Marsh platforms were at the intermediate elevation among the three sites and characterized by a dense cover of both herbaceous (e.g., *Limonium vulgare*) and shrub (e.g., *Atriplex portulacoides*) species. Here, strong positive biotic–abiotic feedbacks were common whereby the dense vegetation cover effectively trapped and stabilized suspended silty and clayey particles during waterlogging, thereby increasing marsh surface elevation. Moreover, individual plants provided organic matter to the marsh floor and ameliorated soil salinity levels [48,52,53]. These, in turn, benefited the vegetation growth. We determined that the platforms represented the early- to mid-biogeomorphic successional stage.

### Statistical procedure for 3B-PLS

In each of the six data sets (three from the Congaree River floodplain and three from the Skallingen salt marsh), vegetation, soil, and topography blocks were of different size; namely, the number of variables in each block was different. For example, the point bar data from Skallingen consisted of seven plant species, 10 soil physical and chemical properties, and two topographic parameters. To scale these varying block sizes (BS) into 1, the following modification was applied for each data value (DV) within each block (adapted from [54]):

$$DV\;\times \;\sqrt{1/BS}$$ (1)

After this data pre-processing, the first stage of 3-B PLS was to perform a principal component analysis (PCA) for each block and extract the first principal components of vegetation, soil, and topography. In this paper, these components were called the starting three unit vectors, one each for vegetation, soil, and topography blocks: *U*_*v*_, *U*_*s*_, and *U*_*t*_, respectively. Treating these unit vectors as linear combinations, we computed:

$$\begin{array}{ll} s_{v} & =X_{v}\;\times \;U_{v}\\ s_{s} & =\;X_{s}\;\times \;U_{s}\\ s_{t} & =\;X_{t}\;\times \;U_{t} \end{array}$$ (2)

where *X*_*v*_, *X*_*s*_, *X*_*t*_ indicated the original vegetation, soil, and topography data blocks, respectively. In other words, *X* was a data matrix with *N* rows (samples) and *k* columns (variables or species) (i.e., *X* = *N* × *k*). Normalizing each *s* to sample variance 1, we then calculated the correlations (*r*) among *s*_*v*_, *s*_*s*_, and *s*_*t*_ in pairs:

$$\begin{array}{l} r_{vs}=\text{correlation}\left(s_{v},\;s_{s}\right)\\ r_{st}=\text{correlation}\left(s_{s},\;s_{t}\right)\\ r_{tv}=\text{correlation}\left(s_{t},\;s_{v}\right) \end{array}$$ (3)

After this first iteration of 3-B PLS, we began the second iteration by updating the original unit vectors, *U*_*v*_, *U*_*s*_, and *U*_*t*_, based on the inter-block correlation structure as follows:

$$\begin{array}{l} U_{v}\;\leftarrow \;X_{v}\left(r_{vs}s_{s}+\;r_{tv}s_{t}\right)\\ U_{s}\;\leftarrow X_{s}\left(r_{st}s_{t}+\;r_{vs}s_{v}\right)\\ U_{t}\;\leftarrow \;X_{t}\left(r_{tv}s_{v}+\;r_{st}s_{s}\right) \end{array}$$ (4)

These new unit vectors represented the summation of “predictions of the individual variables using combinations of the scores *s* with the correlations *r* as weights” ([41], p. 185). We then returned to Eq 2 in order to re-iterate these optimization procedures until we have acquired stable correlation coefficients (*r*_*vs*_, *r*_*st*_, and *r*_*tv*_) that did not vary between iterations (see Fig 5 of [46] for an example). At such convergence, the final vectors, *U*_*v*_, *U*_*s*_, and *U*_*t*_, were designated as the first singular axes representing the data blocks of vegetation, soil, and topography, respectively.

These singular axes are conceptually and statistically distinct from principal components in that a PCA extracts principal components from the *internal* correlation structure within each block of data without considering the covariation across data domains. Each principal component thus maximally represents the overall pattern of only a given data block. In contrast, 3-B PLS yields the singular axes of a data block based on the *weighted combination* of the other two blocks as expressed in Eq 4. Each singular axis should therefore represent the correlations of the elements of a given data block with other blocks.

In this paper, our analysis and results are concerned primarily with the first singular axes that have the greatest mutual correlation among vegetation, soil, and topography blocks. However, if interested, one can also extract the second (and higher) axes by applying the same procedure, explained above, to the residuals after the first singular vectors have been regressed out of the original data blocks. An example Microsoft Excel^®^ implementation of the 3B-PLS procedure is given in the supporting information (see S1 File).

### Estimating bi-variance and tri-variance

**Fig 2:**
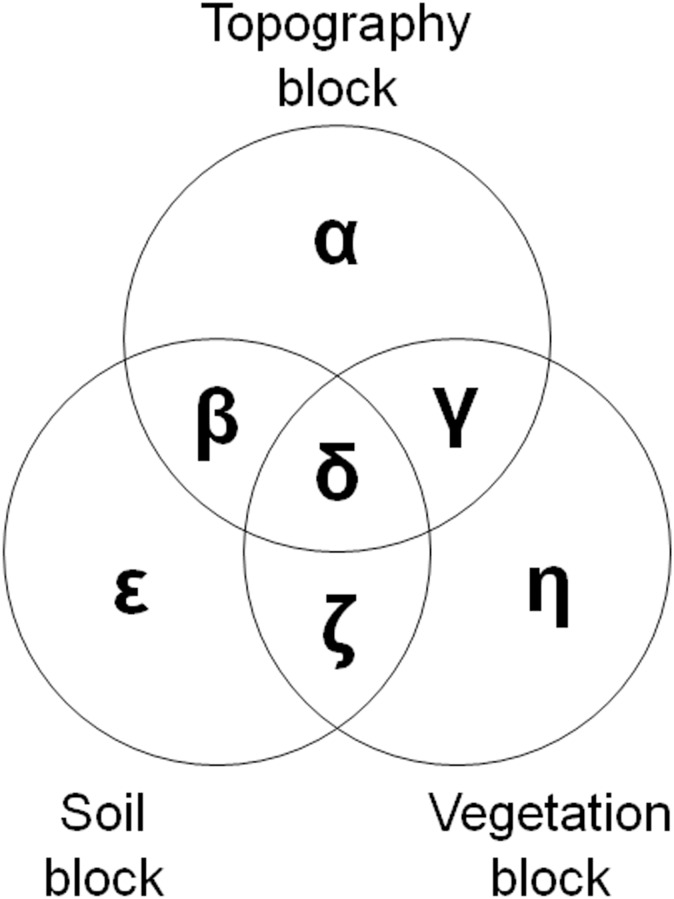
Representation of bi-variance (β+γ+δ+ζ, all in %) and tri-variance (δ, in %) in vegetation–soil–topography relationships. Symbols, α, ε, and η, represent uni-variances. The three circles here are of the same size because the corresponding three singular axes have been standardized (see text).

The strength of vegetation–soil–topography couplings along the FBS gradient was conceptualized and quantified as bi-variance and tri-variance (for more details, see [46]). Bi-variance indicates the amount of the covariation between any two data blocks, that is, between any two singular axes of vegetation, soil, and topography (β+γ+δ+ζ in Fig 2). Tri-variance is the amount of the common variance shared by all of the three singular axes (δ). Lastly, uni-variance is the variance in a data block that is unrelated to the other two blocks (i.e., unexplained variance or “error” in standard parametric statistics; α, ε, and η).

In the biogeomorphic *feedback* framework employed in this research, vegetation, soil, and topography can each be treated as either independent or dependent variables. For example, the unexplained variance of topography (α) was estimated by designating the singular axis of topography as the dependent variable and those of soil and vegetation as the independent variables. To calculate β and γ, we employed the concept of extra sums of squares (ESS) that measured the marginal reduction in the residual sum of squares that occurred due to the addition of an independent variable to the existing regression model [46]. Put another way, if vegetation was already included as an independent variable in a regression model, ESS computed how much additional variation of topography would be explained after adding soil as a new independent variable. This additional variation was equivalent to the pure effect of soil on topography (β), excluding that of vegetation. By switching the role of soil and vegetation (i.e., this time, soil as an existing independent variable in the regression model and vegetation as a newly-added independent variable), ESS calculated γ, or the pure effect of vegetation on topography, excluding soil’s effect. The tri-variance (δ) was then calculated by subtracting α, β, and γ values from the total sums of squares (TSS) of topography. The other variances, ε, ζ, and η, were estimated following the same procedure explained above. Finally, we expressed these raw variance values in proportion (%) to TSS:

Final variance (%)=100 ×(Raw variance/TSS) (5)

All of these procedures were conducted using R 2.14.2 [55]. An example R-code for this procedure is given in the supporting information (see S2 File).

### Constructing Venn diagrams

We characterized the changing bi- and tri-variate relationships among vegetation, soil, and topography along the FBS gradient using Venn diagrams. To best reflect potentially varying values of α through η (Fig 2), we generated *proportional* circular Venn diagrams for each of the six study sites using a statistical loss function and a minimization procedure developed by Wilkinson [56]. This approach has been increasingly used for both exploration and inference on real data sets. We employed an R function venneuler(), which was available as a package in CRAN (www.r-project.org). In each site, the three circles—one each for the singular axis of vegetation, soil, and topography—should be of the same size because the corresponding three singular axes had been standardized before estimating the proportions.

## Results

### General overview

The topographic, edaphic, and vegetational characteristics in each of the floodplain forest and the salt marsh creek sites have already been described in our previous publications (e.g., [47,48]). The new contribution of this present work is discussing the biogeomorphic couplings among these biotic and abiotic factors across within-system succession gradients. The previous publications did not examine such couplings at such a detailed level. In this paper, we provide the detailed information of inter-site physical and biological differences in S1 and S2 Tables. A brief overview follows below.

**Fig 3:**
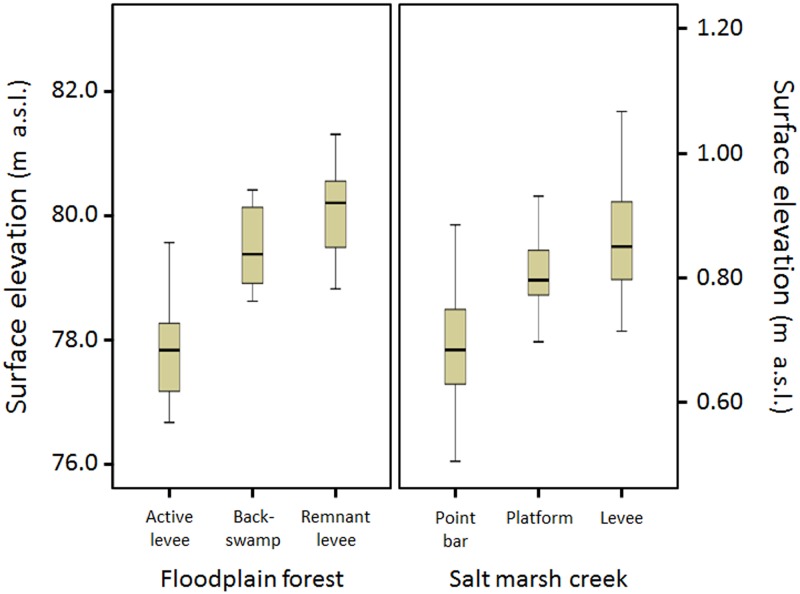
Elevation (m above sea level) range of each site of two study areas (floodplain forest and salt marsh).

Most noticeable and relevant to this research was the significant differentiation of elevation ranges among the three sites of both study areas (Fig 3; *P* < 0.0001 based on the one-way analysis of variance for each study area). In the Congaree River floodplain sites, elevation increased progressively from the active levee to backswamp to remnant levee sites. Reflecting this elevation trend, obligate wetland species were most dominant at the active levee and backswamp sites (e.g., *Quercus lyrata* and *Carya aquatica*), while the remnant levee site contained a much greater component of facultative and facultative wetland species (e.g., *Fraxinus pennsylvanica* and *Platanus occidentalis*; S1 Table).

Similarly, vegetation between sites was highly differentiated across the salt marsh creeks on the basis of elevation and related gradients (S2 Table). The low-lying point bar sites showed much higher occurrence probabilities of pioneer plants (*Puccinellia*, *Suaeda*, *Salicornia*, and *Spartina*) than the platform and levee sites. The bar sites, however, almost lacked high-marsh plants (*Festuca*, *Artemisia*, and *Juncus*), which predominated the natural levee site. The platform site was most diverse in species composition with a high proportion of mid-marsh plants (especially, *Atriplex*) and a lingering presence of pioneers and *Artemisia*.

### Bi-variance and tri-variance along the FBS gradient

**Fig 4:**
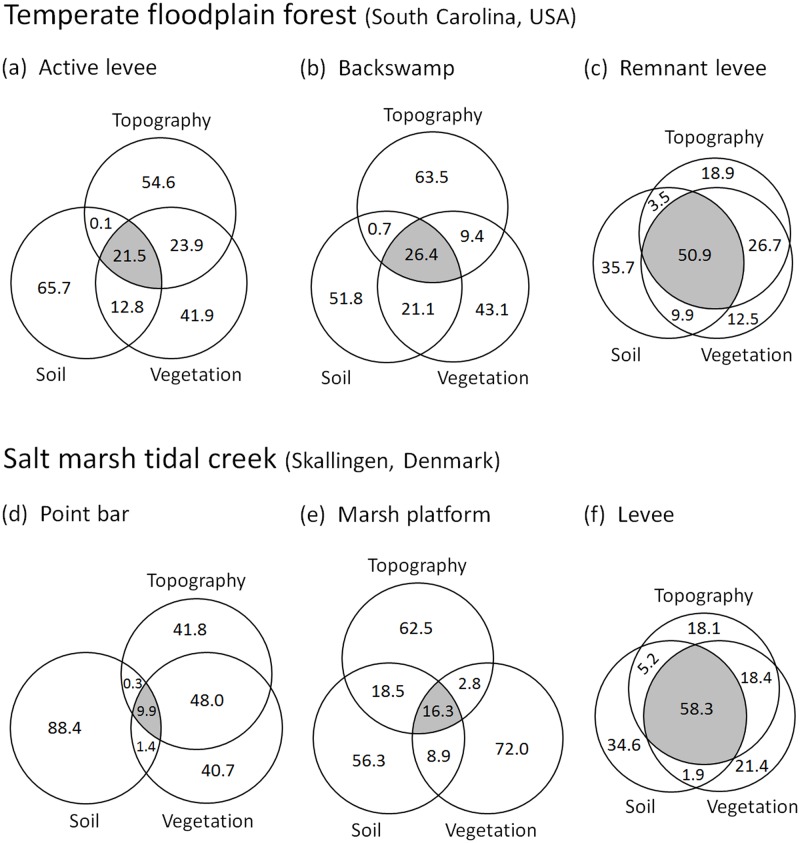
Proportion (%) of variances estimated from the relationships among the first singular axes of vegetation, soil, and topography, identified by 3-B PLS for the floodplain forest data (a–c) and for the salt marsh data (d–f). The three circles of each site are of the same size because the corresponding three singular axes had been standardized before estimating the proportions.

There were striking similarities between the two study areas in trends of bi-variance and tri-variance values along the FBS gradient (Fig 4). Most notably, tri-variance increased from the active levee site (21.5%), to the backswamp site (26.4%), to the remnant levee site (50.9%) in the floodplain forest, and from the point bar site (9.9%), to the platform site (16.3%), to the natural levee site (58.3%) in the marsh creek. This increasing pattern corresponded to that of surface elevation (Fig 3) and the implied gradients of disturbance, mentioned in section **Placing study sites along the FBS gradient**.

Like tri-variance, bi-variance was greatest at the highest-elevation sites: the remnant levee site at the floodplain forest (91.0%) and the marsh levee site at Skallingen (83.8%). The lowest bi-variances, however, were detected at the mid-elevation sites (57.5% at the floodplain backswamp and 46.5% at the marsh platform), not at the lowest-lying sites (58.2% at the active floodplain levee and 59.6% at the marsh point bar). It should be noted that such lowest bi-variance values of the backswamp and marsh platform sites were caused primarily by the very low *pure* bi-variance between topography and vegetation at these sites (9.4% and 2.8%, respectively). At the other four sites, the pure topography–vegetation bi-variance was generally high, at least 18.4% and even up to 48.0%.

## Discussion

The tri-variances estimated from the three sites in each study area support our hypothesis that the strength of vegetation–soil–topography couplings increases along the FBS gradient. This further implies that the recurrence interval of physical disturbance is important to—and potentially serves as an indicator of—the intensity and mechanisms of biotic–abiotic feedback in fluvial biogeomorphic systems. Situated at the lowest elevations, the active levee site along the Congaree River floodplain and the point bar site at the Skallingen marsh undergo the most frequent and intense hydrogeomorphic forcings, which dynamically rework soil substrates, restructure surface landforms, and control the mortality and colonization of plant species. Along the Congaree River, these effects are compounded by high sedimentation rates that constantly reshape soil conditions and act as a stress mechanism on regenerating vegetation. These dynamics foster constant disturbance conditions, under which vegetation, soil, and topography are most likely to show discrete, stochastic, and individualistic behaviors over space and time. Therefore, a loose coupling among the three factors would be expected.

The remnant levee and marsh levee sites, in contrast, exhibit the low recurrence interval (or near-rarity) of disturbance events that may disrupt the existing vegetation–soil–topography relationships there. Hence, ecological succession, soil-forming processes, and landform evolution will occur in tight conjunction with one another over a prolonged time period, thereby maximizing the level of coupling among them. To wit, the three behave *in unity* over space and time. With surprising consistency, all of the six tri-variances calculated in this paper demonstrate that these ideas potentially hold true across the biogeomorphic succession gradient in both coastal and riparian environments.

The lowest bi-variances at the intermediate-elevation sites—caused by the very low pure bi-variance between topography and vegetation at these sites—can be explained given their poorly-draining conditions. In the Congaree River floodplain, the backswamp site soils are classified as poorly-drained loams, whereas those of the active and remnant levee sites are moderately well-drained, silty-clay loams [47,57]. In the salt marsh area, the platform site consists primarily of silty and clayey materials, thereby delaying the percolation of sea water during ebb tides, but the point bar and levee sites are composed mostly of sandy substrates, inducing a rapid percolation process [58,59]. Within poorly-drained locations, such as those at the backswamp and marsh platform sites, hydrologic factors that are critical to plant growth likely vary over space, in little conjunction with micro-scale topographic relief patterns (e.g., low difference in moisture between local mounds and depressions due to delayed percolation). This would potentially reduce the strength of the correlation between topography and vegetation. If the backswamp and marsh platform sites had been well-drained as in the other four sites, much tighter topography–vegetation couplings would likely have been detected because the submergence regime would more closely track the ambient topographic variations. Then, such increased correlations between topography and vegetation could have augmented the overall bi-variances of these mid-elevation sites, possibly beyond those of the lowest-lying sites (i.e., the active levee and point bar).

Our results—in particular, those related to changing biotic–abiotic relationships along successional and disturbance gradients—support previous studies that have documented the greater importance of stochastic processes during initial successional stages (e.g., [60]) and bear an important implication for species distribution modeling (SDM) in future research [61–65]. Previous SDM studies have often postulated, or even taken for granted, the existence of significant couplings between community structure and environmental factors, thereby explicitly used soil and topographic attributes as independent variables for predicting species distribution. However, we suggest avoiding heavy reliance on such independent variables during relatively early or retarded stages of biogeomorphic succession when a system is highly prone to external hydrodynamic pulsing and disturbance. Under such conditions, seedling recruitment and soil-forming processes are highly stochastic [7,37], and results of SDM are likely to be spurious, rather than representing real causality in vegetation–environment relationships [66–68].

Indeed, this same argument was made by Kim and Arthur [69], who posited that special care should be taken when performing SDM “immediately after a fire disturbance or where disturbance has a high frequency” (p. 669). In an oak-dominated temperate forest of eastern Kentucky, USA, Kim and Arthur [69] investigated how the strength of tree species–environment couplings varied in response to recurring prescribed fire events (2–4 times) across 32 plots from 2002 and 2010. They found that, before 2002, the forests had maintained close biotic–abiotic relationships in the long absence of major fires for more than 30 years. Since 2002, however, a dramatic weakening of such relationships has been observed during and immediately after the periods of fire disturbance. Furthermore, after the devastative eruption of Mount St. Helens, Washington, USA in 1980, researchers found little correlation of recovering vegetation with environment in the early stages of primary succession, presumably because stochastic recruitment and chance survival were playing the leading roles [70,71]. All of these empirical, consistent evidences lead us to generalize that patterns of vegetation–environment relationships are contingent upon the magnitude, frequency, and timing of disturbance during the course of system dynamics over a wide range of ecological settings (e.g., river floodplain, tidal marsh creek, mountain forest, and post-volcanism regenerative slope; see also [46,72–74]). We propose that bi-variance and tri-variance provide useful insights into changing relationships among different data domains across scales and disturbance regimes.

We recognize that all successional pathways do not necessarily follow the linear, four-phase sequence described in the FBS model (Fig 1). Rather, a more realistic perspective would involve multiple-pathways that account for the possibility of nonlinear, stochastic adjustments of a fluvial biogeomorphic system responding to, for example, changes in discharge, erosion, and in-stream accumulation of large woody debris [75–79]. We, however, still believe that the original FBS is a useful simplification of the actually complex, multi-faceted nature of fluvial landscape dynamics (see [10,23]) and that the model will therefore serve as an important springboard for better understanding and predicting varying patterns of vegetation–soil–topography relationships over time.

In our three-block Venn diagram approach, it does not matter whether one designates vegetation, soil, and topography as dependent or independent variables. That is, the approach treats the three components at the same level and, hence, it is symmetrical. We posit that this idea closely accords with an ever-increasing perspective in ecology: recursive feedbacks among biological, edaphic, and terrain factors. Traditionally, many ecological studies have been predominantly concerned with one-way relationships of vegetation, soil, and topography, focusing on the influence of one factor upon another. Such a unidirectional logic necessitated a division of these components into groups of dependent and independent variables, followed by the application of typical asymmetrical methods, like multiple regression and direct gradient analysis [80–83]. Recently, recognizing the importance of the reciprocal nature of vegetation–soil–topography linkages (see Introduction), many ecologists have redirected their attention from discrete one-way couplings to integrated two- or three-way interactions [1,4,11,13,40]. Despite this paradigm shift, we still seem to be methodologically constrained by conventional multivariate statistics, and face a challenge regarding how to divide our data into groups of dependent and independent variables. Our approach avoids such a mono-directional assumption structure by presenting Venn diagrams that fully reflect the idea of mutual feedbacks among vegetation, soil, and topography at the same level in a range of fluvial biogeomorphic systems. A conceptual and methodological shift like we are attempting in this paper will be increasingly important as ecology grows even more integrative.

## Supporting Information

S1 Fig Congaree River floodplain area. (a) Geographic location of the floodplain sites at Congaree National Park, South Carolina, USA. The small rectangular box indicates (b), in which 32 sample transects (yellow lines) were located in three different topographic settings (active levee, remnant levee, backswamp) at the Bates Fork Tract. (c) Sampling vegetation in a 2.82 m radius circular subplot at one of the backswamp sites. (d) Field design for locating sample sub-plots along a 50 m-transect (thick, black arrow) extending from the forest edge into early regrowth in clear cuts. Sub-plots associated with Plots 1 and 2 were located 5–10 m and 30–50 m into the clear cut, respectively.
(PDF)

S2 Fig Salt marsh creek area. (a) Geographic location of the Skallingen salt marsh in Denmark. The small rectangular box indicates (b) in which 11 point bars of this research are situated. (c) One of the 11 bars (P8). (d) Field design for sampling vegetation, soil, and topography across each of the 11 bars.
(PDF)

S1 File Procedure for 3B-PLS. (XLS)

S2 File Procedure for estimating extra sums of squres. (TXT)

S1 Table Average values of surface elevation, inundation depth, soil properties, and selected plant species abundance (18 most common only; basal area, cm^2^ ha^–1^) at each study site in the Bates Fork tract of Congaree National Park, South Carolina, USA. (PDF)

S2 Table Average values of surface elevation, distance to the creek, soil properties, and all plant species abundance (% frequency) at each study site of the Skallingen salt marsh, Denmark. (PDF)

S1 Text Study areas and data collection. Description of the areas studied (temperate floodplain and salt marsh creek) and field methods.
(PDF)
