# Supplementary material for: Tri-Variate Relationships among Vegetation, Soil, and Topography along Gradients of Fluvial Biogeomorphic Succession
Source: PLoS One. 2016 Sep 20;11(9):e0163223. doi: 10.1371/journal.pone.0163223 (PMC5029874; doi:10.1371/journal.pone.0163223)
Supplement: S1 Fig — (a) Geographic location of the floodplain sites at Congaree National Park, South Carolina, USA. The small rectangular box indicates (b), in which 32 sample transects (yellow lines) were located in three different topographic settings (active levee, remnant levee, backswamp) at the Bates Fork Tract. (c) Sampling vegetation in a 2.82 m radius circular subplot at one of the backswamp sites. (d) Field design for locating sample sub-plots along a 50 m-transect (thick, black arrow) extending from the forest edge into early regrowth in clear cuts. Sub-plots associated with Plots 1 and 2 were located 5–10 m and 30–50 m into the clear cut, respectively. (PDF) [file pone.0163223.s001.pdf]

Supporting Information

Tri-variate relationships among vegetation, soil, and topography along the gradient of fluvial biogeomorphic succession

Daehyun Kim · John A. Kupfer

S1 Fig. Congaree River floodplain area. (a) Geographic location of the floodplain sites at Congaree National Park, South Carolina, USA. The small rectangular box indicates (b), in which 32 sample transects (yellow lines) were located in three different topographic settings (active levee, remnant levee, backswamp) at the Bates Fork Tract. (c) Sampling vegetation in a 2.82 m radius circular subplot at one of the backswamp sites. (d) Field design for locating sample sub-plots along a 50 m-transect (thick, black arrow) extending from the forest edge into early regrowth in clear cuts. Sub-plots associated with Plots 1 and 2 were located 5-10 m and 30-50 m into the clear cut, respectively.

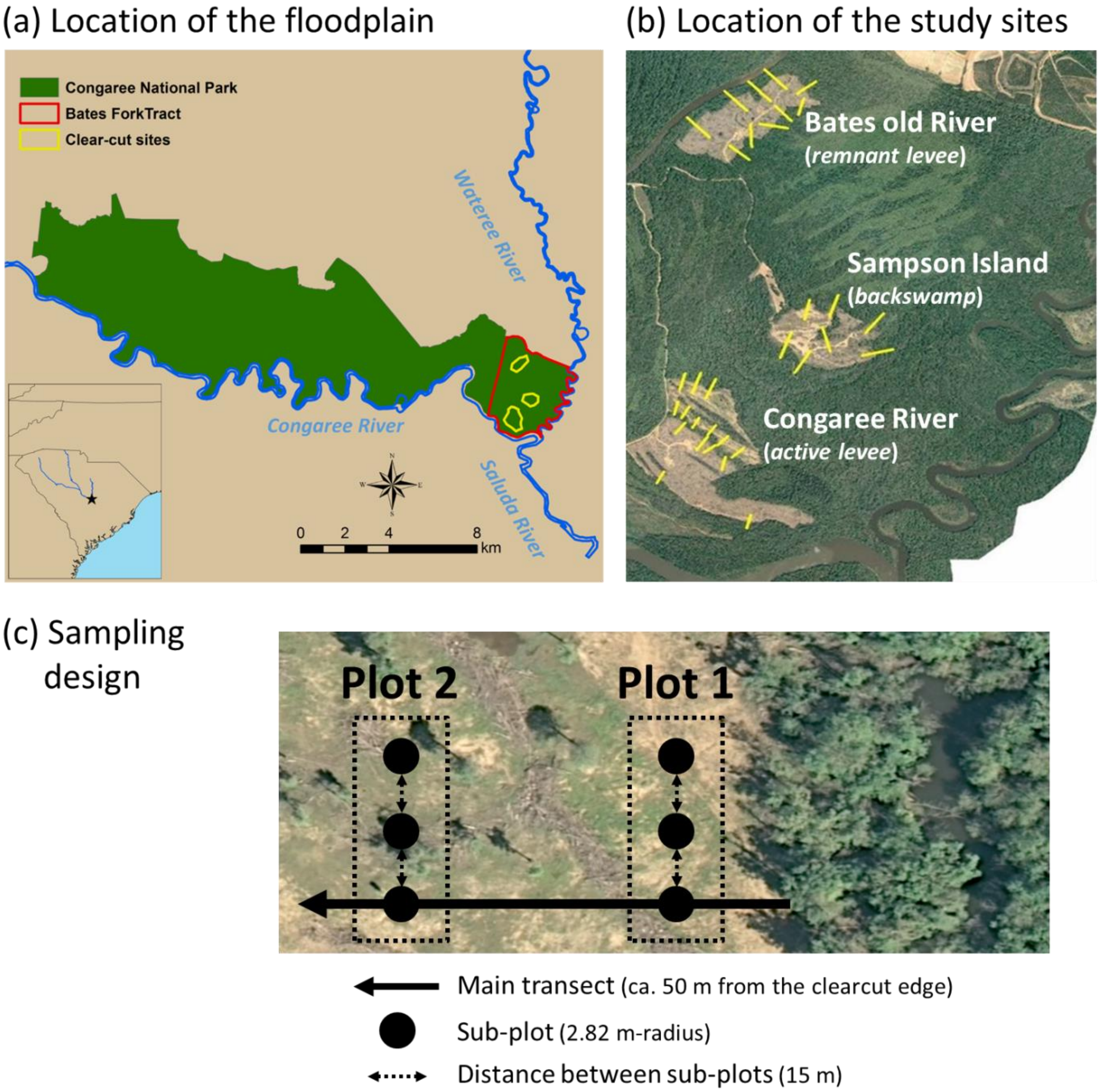
(a) Location of the floodplain
Congaree National Park
Bates ForkTract
Clear-cut sites
Wateree River
Congaree River
Saluda River
0
2
4
8
km
(b) Location of the study sites
Bates old River
(remnant levee)
Sampson Island
(backswamp)
Congaree River
(active levee)
(c) Sampling design
Plot 2
Plot 1
Main transect (ca. 50 m from the clearcut edge)
Sub-plot (2.82 m-radius)
Distance between sub-plots (15 m)
