# Supplementary material for: Tri-Variate Relationships among Vegetation, Soil, and Topography along Gradients of Fluvial Biogeomorphic Succession
Source: PLoS One. 2016 Sep 20;11(9):e0163223. doi: 10.1371/journal.pone.0163223 (PMC5029874; doi:10.1371/journal.pone.0163223)

## Supporting Information

# Tri-variate relationships among vegetation, soil, and topography along the gradient of fluvial biogeomorphic succession

**Daehyun Kim · John A. Kupfer**

**S2 Fig. Salt marsh creek area.** (a) Geographic location of the Skallingen salt marsh in Denmark. The small rectangular box indicates (b) in which 11 point bars of this research are situated. (c) One of the 11 bars (P8). (d) Field design for sampling vegetation, soil, and topography across each of the 11 bars.

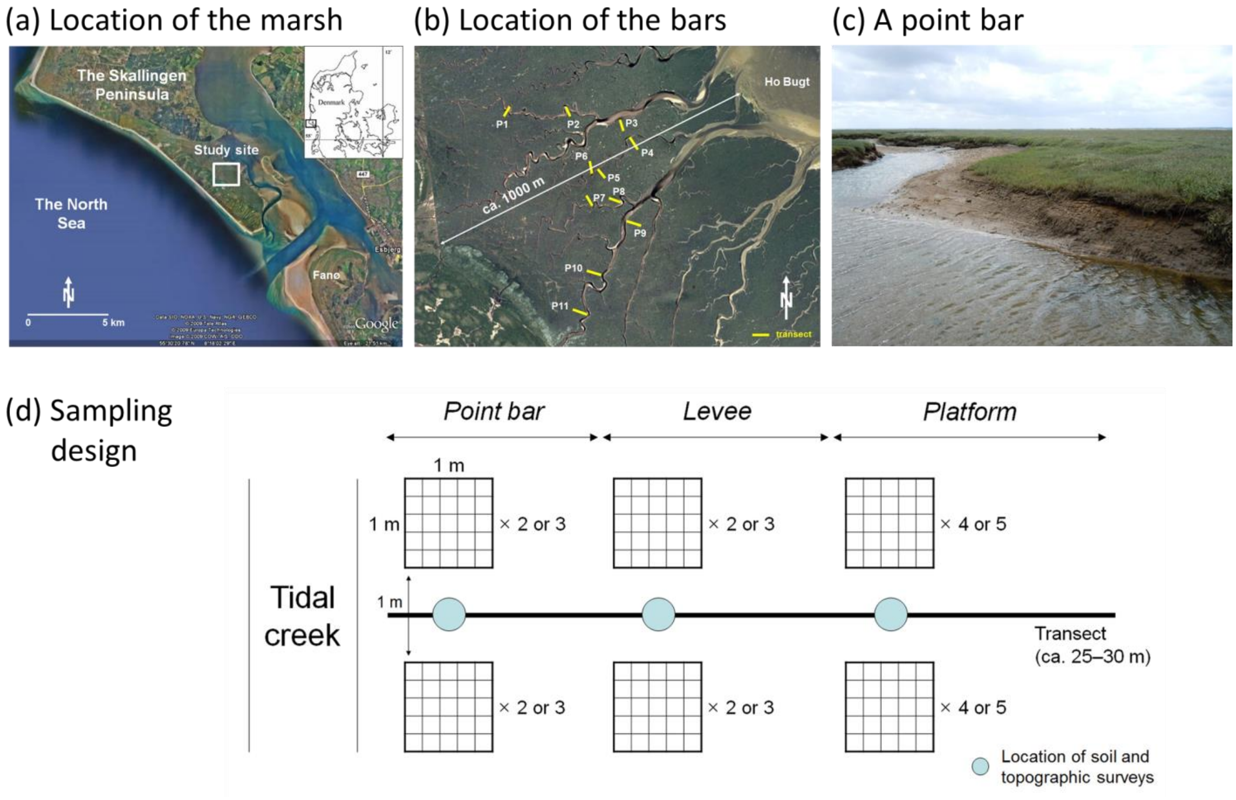

Supplement: S2 Fig — (a) Geographic location of the Skallingen salt marsh in Denmark. The small rectangular box indicates (b) in which 11 point bars of this research are situated. (c) One of the 11 bars (P8). (d) Field design for sampling vegetation, soil, and topography across each of the 11 bars. (PDF) [file pone.0163223.s002.pdf]
