# Supplementary material for: Tri-Variate Relationships among Vegetation, Soil, and Topography along Gradients of Fluvial Biogeomorphic Succession
Source: PLoS One. 2016 Sep 20;11(9):e0163223. doi: 10.1371/journal.pone.0163223 (PMC5029874; doi:10.1371/journal.pone.0163223)
Supplement: S1 Table — (PDF) [file pone.0163223.s005.pdf]

Supporting Information

Tri-variate relationships among vegetation, soil, and topography along the gradient of fluvial biogeomorphic succession

Daehyun Kim · John A. Kupfer

S1 Table. Average values of surface elevation, inundation depth, soil properties, and selected plant species abundance (18 most common only; basal area, $cm^2\ ha^{-1}$) at each study site in the Bates Fork tract of Congaree National Park, South Carolina, USA.

	Active levee (n[a] = 31)	Backswamp (n = 14)	Remnant levee (n = 18)	F-value[b]
Surface elevation (m)	77.9 ± 0.8[c]	79.5 ± 0.6	80.3 ± 1.0	51.79***
Depth of inundation[d] (m)	9.6 ± 0.9	7.3 ± 0.7	8.4 ± 0.9	36.06***
Soil pH	4.9 ± 0.1	5.0 ± 0.1	4.9 ± 0.1	3.68*
P ($mg\ kg^{-1}$)	5.2 ± 1.1	6.4 ± 1.7	5.6 ± 1.5	3.70*
K ($mg\ kg^{-1}$)	62.3 ± 11.9	78.3 ± 23.4	68.1 ± 20.0	4.08*
Ca ($mg\ kg^{-1}$)	804.9 ± 102.4	932.9 ± 187.2	938.7 ± 155.3	6.90**
Mg ($mg\ kg^{-1}$)	239.7 ± 40.0	255.3 ± 47.8	242.7 ± 33.5	0.74
Zn ($mg\ kg^{-1}$)	3.8 ± 1.1	4.8 ± 1.3	5.5 ± 1.4	10.40***
Mn ($mg\ kg^{-1}$)	89.7 ± 24.3	85.5 ± 37.0	98.8 ± 19.9	1.10
Cu ($mg\ kg^{-1}$)	4.0 ± 0.5	3.9 ± 0.8	4.2 ± 0.7	1.02
Na ($mg\ kg^{-1}$)	45.5 ± 11.1	41.3 ± 7.9	33.9 ± 6.9	8.58**
Organic matter (%)	5.9 ± 1.0	6.6 ± 1.0	5.9 ± 0.8	2.70

Cation exchange capacity (mg kg^{-1})	9.1 ± 0.4	9.6 ± 0.9	9.0 ± 0.5	5.51**
Acidity	6.0 ± 0.4	6.1 ± 0.3	5.5 ± 0.3	14.19***
Base saturation (%)	34.8 ± 3.7	36.9 ± 4.2	38.9 ± 3.9	6.41**
Quercus lyrata (O-W[e])	6.8 ± 27.2	1.5 ± 3.2	0.7 ± 1.8	0.70
Nyssa species (O-W)	5.7 ± 31.7	0.1 ± 0.3	0.0 ± 0.0	0.50
Carya aquatica (O-W)	4.5 ± 23.0	8.9 ± 29.8	0.1 ± 0.3	0.68
Populus species (O-W)	3.4 ± 10.6	1.9 ± 4.9	0.7 ± 2.2	0.70
Planera aquatica (O-W)	1.4 ± 4.2	4.1 ± 7.6	0.0 ± 0.0	3.16*
Salix nigra (O-W)	0.2 ± 0.9	7.8 ± 25.0	0.0 ± 0.0	2.40
Taxodium distichum (O-W)	0.1 ± 0.5	28.2 ± 102.6	0.1 ± 0.4	1.88
Fraxinus pennsylvanica (F-W)	30.8 ± 61.1	1.3 ± 2.4	68.8 ± 62.6	6.21**
Ilex decidua (F-W)	9.0 ± 20.6	1.5 ± 3.6	0.3 ± 0.8	2.43
Quercus laurifolia (F-W)	3.1 ± 6.1	0.5 ± 1.3	4.5 ± 7.9	1.71
Platanus occidentalis (F-W)	0.0 ± 0.0	0.0 ± 0.0	4.2 ± 15.2	1.73
Ulmus americana (F-W)	0.6 ± 1.6	2.0 ± 5.6	2.9 ± 6.2	1.63
Celtis laevigata (F-W)	0.2 ± 0.8	1.6 ± 3.7	1.8 ± 6.2	1.24
Liquidambar styraciflua (F)	17.9 ± 26.1	49.8 ± 117.7	11.1 ± 18.8	1.94
Acer rubrum (F)	9.3 ± 37.8	0.0 ± 0.0	10.2 ± 19.6	0.62
Rubus species (F)	20.6 ± 46.1	0.5 ± 0.9	5.2 ± 5.8	2.32
Diospyros virginiana (F)	0.3 ± 1.5	0.2 ± 0.6	5.0 ± 18.7	1.47
Quercus nigra (F)	0.0 ± 0.0	0.4 ± 1.5	2.4 ± 9.4	1.28

[a] number of plots in the corresponding site

[b] estimated by one-way analysis of variance

[c] standard deviation

[d] during a 98,000 cfs flood

[e] United States Fish and Wildlife Service Regional Wetland Indicator groups (O-W = obligate wetland species, F-W = facultative wetland species, F = facultative species)

*** significant at the level of 0.1% probability (two-tailed; $P < 0.001$)

** significant at the level of 1% probability (two-tailed; $P < 0.01$)

* significant at the level of 5% probability (two-tailed; $P < 0.05$)
