# Supplementary material for: Tri-Variate Relationships among Vegetation, Soil, and Topography along Gradients of Fluvial Biogeomorphic Succession
Source: PLoS One. 2016 Sep 20;11(9):e0163223. doi: 10.1371/journal.pone.0163223 (PMC5029874; doi:10.1371/journal.pone.0163223)
Supplement: S2 Table — (PDF) [file pone.0163223.s006.pdf]

Supporting Information

Tri-variate relationships among vegetation, soil, and topography along the gradient of fluvial biogeomorphic succession

Daehyun Kim · John A. Kupfer

S2 Table. Average values of surface elevation, distance to the creek, soil properties, and all plant species abundance (% frequency) at each study site of the Skallingen salt marsh, Denmark.

	Point bar (n^a = 26)	Platform (n = 41)	Levee (n = 35)	F-value[b]
Surface elevation (m DNN[c])	0.7 ± 0.1[d]	0.8 ± 0.1	0.9 ± 0.1	28.96***
Distance to the creek (m)	1.7 ± 1.2	18.7 ± 6.3	6.9 ± 2.7	140.47***
Soil pH	6.9 ± 0.4	6.0 ± 0.4	6.4 ± 0.5	36.50***
Bulk density (g cm^{-3})	1.0 ± 0.3	0.8 ± 0.2	0.9 ± 0.3	8.77***
EC[e] (umhos cm^{-1})	7499.2 ± 1904.6	9344.1 ± 1227.5	7978.6 ± 2827.5	7.48**
Nitrate (mg kg^{-1})	19.5 ± 29.8	106.5 ± 101.0	110.1 ± 99.9	9.80***
P (mg kg^{-1})	84.1 ± 29.1	105.8 ± 65.5	96.3 ± 45.4	1.42
K (mg kg^{-1})	583.3 ± 313.3	1007.5 ± 348.6	786.5 ± 387.2	11.71***
Ca (mg kg^{-1})	2499.2 ± 980.5	1373.8 ± 371.9	1502.6 ± 657.3	24.98***
Mg (mg kg^{-1})	1172.6 ± 596.3	2095.6 ± 691.8	1691.2 ± 807.3	13.42***
S (mg kg^{-1})	740.3 ± 404.0	1158.9 ± 501.3	964.9 ± 507.6	6.08**
Na (mg kg^{-1})	7482.5 ± 3970.6	11731.8 ± 4129.3	9154.0 ± 4636.4	8.41***

Puccinellia maritima (P[f])	63.9 ± 30.7	52.9 ± 21.3	22.7 ± 21.1	25.54***
Suaeda maritima (P)	84.4 ± 23.4	25.8 ± 28.5	16.2 ± 24.1	59.03***
Salicornia herbacea (P)	59.9 ± 36.9	18.0 ± 28.4	4.3 ± 10.1	34.45***
Spartina anglica (P)	20.1 ± 25.5	1.5 ± 4.8	0.5 ± 1.6	20.32***
Spergularia media (M)	0.0 ± 0.0	1.0 ± 3.4	0.3 ± 1.2	1.90
Aster tripolium (M)	18.2 ± 16.5	22.7 ± 19.2	5.1 ± 7.8	12.71***
Limonium vulgare (M)	32.9 ± 19.4	47.5 ± 26.4	15.7 ± 19.2	18.92***
Triglochin maritima (M)	0.0 ± 0.0	13.4 ± 17.5	2.2 ± 6.7	13.36***
Plantago maritima (M)	0.9 ± 2.3	21.7 ± 22.0	7.9 ± 10.1	16.33***
Atriplex portulacoides (M)	36.2 ± 33.1	99.0 ± 4.7	87.7 ± 28.0	59.96***
Festuca rubra (H)	0.1 ± 0.4	0.0 ± 0.3	21.0 ± 35.9	11.39***
Artemisia maritima (H)	1.5 ± 4.4	8.6 ± 22.2	36.2 ± 35.8	16.97***
Juncus gerardii (H)	0.0 ± 0.0	0.3 ± 1.4	22.9 ± 38.7	11.51***

[a] number of plots in the corresponding site

[b] estimated by one-way analysis of variance

[c] Danish Ordnance Zero

[d] standard deviation

[e] electrical conductivity

[f] general classification of the species at Skallingen based on each species's elevation ranges and successional stages (Kim 2014; Kim *et al*. 2012; P = pioneer plants, M = mid-marsh plants, H = high-marsh plants)

*** significant at the level of 0.1% probability (two-tailed; $P < 0.001$)

** significant at the level of 1% probability (two-tailed; $P < 0.01$)

* significant at the level of 5% probability (two-tailed; $P < 0.05$)
