# Supplementary material for: Tri-Variate Relationships among Vegetation, Soil, and Topography along Gradients of Fluvial Biogeomorphic Succession
Source: PLoS One. 2016 Sep 20;11(9):e0163223. doi: 10.1371/journal.pone.0163223 (PMC5029874; doi:10.1371/journal.pone.0163223)
Supplement: S1 Text — Description of the areas studied (temperate floodplain and salt marsh creek) and field methods. (PDF) [file pone.0163223.s007.pdf]

Supporting Information

Tri-variate relationships among vegetation, soil, and topography along the gradient of fluvial biogeomorphic succession

Daehyun Kim · John A. Kupfer

S1 Text. Study areas and data collection

Temperate floodplain

The Congaree River floodplain data were collected at the 969 ha Bates Fork tract within Congaree National Park (NP) (33°45'24.93"N, 80°37'33.17"W); S1 Fig). The data come from three forested stands that were clear-cut shortly before the tract's acquisition in 2005. Sample sites encompass a gradient of conditions associated with differences in elevation, hydroperiod, geomorphic setting, and soil conditions.

Data were collected using a replicated sample design with sample plots located along 32 transects spanning the boundary between logged and unlogged forest [1]. Along each transect, a circular subplot (2.82 m radius) was randomly located: (1) 5-10 m into the clearcut (field edge), and (2) 30–50 m into the clearcut (field interior). Two additional circular subplots were then established at distances 15 m and 30 m from the initial plot but perpendicular to the main transect line. The diameter and species of all individual woody plants > 1 cm in diameter at breast height (saplings) were measured, while individuals > 0.25 m in height but < 1 cm in dbh

(seedlings) were counted by species. A single measure of species abundance for each subplot was calculated by: (1) transforming sapling diameters to basal areas and summing the values by species, and (2) assigning each seedling a nominal basal area of 0.1 cm^2 and adding these values to the sapling basal area. Values for the three subplots at each transect location were then aggregated to create 'plot-level' data for 64 plots (two plots each at 32 transects).

Soil samples were taken from the top 10-15 cm of soil at four locations within each of the three subplots; these samples were composited to provide data on plot-level soil conditions [1]. All soil samples were analyzed for pH, extractable phosphorus, potassium, calcium, magnesium, zinc, copper and sodium, organic matter, cation exchange capacity(CEC), exchangeable acidity (the amount of total CEC occupied by H^+ and Al^{3+}) and total percent base saturation (the percent of exchange sites occupied by base cations). Additionally, two measures associated with site topography and flooding for each plot were determined: 1) elevation (m asl), which was extracted for each of the circular subplots from a digital terrain model constructed using high-resolution Lidar data with 3 m horizontal accuracy and 10 cm vertical accuracy, and 2) inundation depths for each subplot during a 98,000 cfs flood, a discharge that represents a flood which inundates all of the Bates Fork floodplain. These later values were derived by coupling the U.S. Army Corps of Engineers Hydrologic Engineering Center-River Analysis System (HEC-RAS v.3.1.1: [2]) with a Geographic Information System (ArcGIS 9.2), using a specialized set of ArcGIS extension tools (HEC-GeoRAS v.4.1.9.2: [3]). Sub-plot values for elevation and inundation depth were then averaged to get plot-level values that were consistent with the vegetation and soils data. Additional details concerning field sampling and inundation modeling can be found in Kupfer *et al*. [1].

Salt marsh creek

The study marsh lies within the Skallingen peninsula, which is at the northern end of the Wadden Sea (55°30′29.85″ N, 8°15′03.08″ E; S2 Fig). The marsh is characterized by a micro-tidal condition with the mean range being ca. 1.5 m. The range becomes about 1.7 m at spring and 1.3 m at neap tides. The marsh has formed since the beginning of the 20th century when the accretion of silt and clay began on top of the peninsula's centuries old extensive sand flat [4].

Along each transect, Kim established 1 × 1 m square quadrats, each of which was subdivided into 25 grids of 0.2 m × 0.2 m (S2 Fig). Kim increased the number of quadrats and the distance between them as he moved from the point bar toward the interior (i.e., marsh platform): (1) two or three quadrats were separated by 0.5 m in the zone of point bar, (2) two or three quadrats were separated by 1 m in the natural levee, and (3) four or five quadrats were separated by 2–4 m in the marsh interior. At every site surveyed, two replicate quadrats were established. The presence of vascular plant species was examined in each grid (0.2 m × 0.2 m); therefore, the frequency of each species in one quadrat ranged between 0 and 25. Then, the frequencies from two replicate quadrats were averaged.

At the middle between the two replicate quadrats, Kim conducted topographic and soil surveys. For the topographic survey, he used a differential Global Positioning System (Trimble R4 GPS Receiver and Trimble Recon® Controller). The machine's horizontal and vertical precision was 1 cm and 2 cm, respectively. Distance (m) was measured from each point to the streamline. For the soil sampling, he used a cylindrical core with diameter and depth being 4.5 cm and 10 cm, respectively. A total of 10 soil physical and chemical properties were then analyzed on each soil sample, including bulk density, soil pH, electrical conductivity (umhos cm^{-1}), phosphorus (mg kg^{-1}), sulfur (mg kg^{-1}), nitrate (NO_3^-; mg kg^{-1}), Na^+ (mg kg^{-1}), K^+ (mg kg^{-1}),

Ca^{2+} (mg kg^{-1}), and Mg^{2+} (mg kg^{-1}). See Kim *et al*. ([5]) for more detailed information of analytic procedures.

References

1. Kupfer JA, Meitzen KM, Pipkin AR. Hydrogeomorphic controls of early post-logging successional pathways in a southern floodplain forest. Forest Ecol Manag. 2010; 259: 1880–1889. doi:10.1016/j.foreco.2010.01.050

2. USACE (United Stated Army Corps of Engineers). HEC-RAS, River Analysis System User's Manual. Davis: U.S. Army Corps of Engineers, Hydrologic Engineering Center; 2002.

3. USACE (United Stated Army Corps of Engineers). HEC-GeoRAS: GIS Tools for support of HEC-RAS using ArcGIS. Davis: U.S. Army Corps of Engineers, Hydrologic Engineering Center; 2005.

4. Aagaard T, Nielsen N, Nielsen J. Skallingen – origin and evolution of a barrier spit. Copenhagen: Meddelelser fra Skalling-Laboratoriet. Bind 35; 1995.

5. Kim D, Cairns DM, Bartholdy J, Morgan CLS. Scale-dependent correspondence of floristic and edaphic gradients across salt marsh creeks. Ann Assoc Am Geogr. 2012; 102: 276–294. doi:10.1080/00045608.2011.620520
